# Role of Immunotherapy for Oncogene-Driven Non-Small Cell Lung Cancer

**DOI:** 10.3390/cancers10080245

**Published:** 2018-07-27

**Authors:** Yosuke Miura, Noriaki Sunaga

**Affiliations:** Department of Respiratory Medicine, Gunma University Graduate School of Medicine, 3-39-15, Showa-machi, Maebashi 371-8511, Japan; m15702011@gunma-u.ac.jp

**Keywords:** non-small cell lung cancer, immunotherapy, immune checkpoint inhibitor, oncogenic driver, epidermal growth factor receptor (EGFR), v-Ki-ras2 Kirsten rat sarcoma viral oncogene homolog (KRAS), programmed death ligand-1 (PD-L1), tumor microenvironment.

## Abstract

The clinical application of immune checkpoint inhibitors (ICIs) has led to dramatic changes in the treatment strategy for patients with advanced non-small cell lung cancer (NSCLC). Despite the observation of improved overall survival in NSCLC patients treated with ICIs, their efficacy varies greatly among different immune and molecular profiles in tumors. Particularly, the clinical significance of ICIs for oncogene-driven NSCLC has been controversial. In this review, we provide recent clinical and preclinical data focused on the relationship between oncogenic drivers and immunological characteristics and discuss the future direction of immunotherapy in NSCLC patients harboring such genetic alterations

## 1. Introduction

Lung cancer is the leading cause of cancer-related death worldwide [1]. Non-small cell lung cancer (NSCLC) accounts for almost 85% of all lung cancer cases [2]. The identification of driver oncogenes has provided clinical implications for molecular targeted therapy in NSCLC, particularly for patients harboring *EGFR* activating mutations [3,4,5,6], *BRAF V600E* mutations [7], and oncogenic rearrangements in *ALK* [8,9,10,11,12,13], *ROS1* [13,14] and *RET* [15,16]. However, most patients ultimately develop acquired resistance, which remains challenging to conquer.

The recent development of immune-oncology has drastically changed NSCLC treatment. The receptors of anti-program death 1 (PD-1) and anti-cytotoxic T lymphocyte antigen-4 (CTLA-4) are expressed on activated T cells that normally downregulate excessive immune responses, while tumor cells and tumor-infiltrating immune cells express programmed death-ligand 1 (PD-L1) to evade antitumor immunity through the interaction with these molecules [17]. Immune checkpoint inhibitors (ICIs) targeting PD-1, PD-L1 and CTLA-4 have an increasing role in the treatment of NSCLC [18,19,20,21,22,23,24,25,26,27]. Notably, a recent study showed that the estimated five-year overall survival (OS) rate was 16% for patients with pretreated, advanced NSCLC receiving nivolumab, an anti-PD-1 antibody [28]. However, only a few patients benefit from a durable response to ICIs, and nearly half of patients develop early disease deterioration. Thus, predictive biomarkers for ICIs are eagerly needed. Recent studies have indicated PD-L1 expression, tumor-infiltrating lymphocytes (TILs), tumor mutational burden, neoantigens, and DNA mismatch repair (MMR) deficiency as candidate biomarkers for ICIs. Nevertheless, the predictive significance of these factors remains controversial.

Several phase III trials have demonstrated that ICIs significantly prolong the OS of NSCLC patients compared with chemotherapy [18,19,24]. On the other hand, subset analyses reveal that the treatment efficacy of ICIs varies depending on the type of oncogenic driver. Additionally, few clinical trials have uncovered whether immunotherapy is more effective than molecularly targeted agents or chemotherapy for oncogenic-driven NSCLC patients. Therefore, investigating the immunologic features of oncogene-driven NSCLC is crucial for advances in immunotherapy in these populations.

Among oncogenic drivers for NSCLC, *KRAS* is one of the most frequently mutated proto-oncogenes [29]. Because of the biological diversity of *KRAS*-mutant NSCLC [30,31] and the clinical unavailability of direct inhibitors of KRAS, a treatment strategy for patients with *KRAS*-mutated NSCLC has not yet been established. Given the unfavorable prognosis in *KRAS*-mutant NSCLC patients [32,33], the development of immunotherapy for such populations is highly anticipated. Recent efforts to investigate the underlying immunologic background have provided some indications for immunotherapy in *KRAS*-mutant NSCLC.

In this review, we address the clinical data of immunotherapy and preclinical evidence concerning immunologic characteristics in oncogene-driven NSCLC and discuss future directions for immunotherapy in patients with oncogene-driven NSCLC.

## 2. Biomarkers for Immunotherapy

### 2.1. PD-L1

PD-L1, a logical biomarker to predict the response to anti-PD-1/PD-L1 blockade, has been extensively evaluated in clinical studies. PD-L1 was regarded as a predictive biomarker in a first-in-human study of nivolumab, and a subsequent larger-scale phase I study of NSCLC further confirmed this finding; at a cut-off of 5% PD-L1-positive tumor cells, the overall response rate (ORR) of PD-L1-positive patients was 36%, whereas the ORR of PD-L1-negative patients was 0% [34]. In a phase III trial, CheckMate-057, nivolumab was associated with a longer progression-free survival (PFS) and OS than docetaxel in pretreated non-squamous (non-Sq) NSCLC patients exhibiting high PD-L1 expression [18]. The phase III KEYNOTE-024 trial also demonstrated that pembrolizumab was significantly associated with longer PFS and OS than platinum-based chemotherapy in patients with PD-L1 expression in at least 50% of tumor cells [20]. Based on these results, PD-L1 expression is currently utilized for the indication of anti-PD-1 treatment in advanced NSCLC patients in the clinic. Alternatively, patients with PD-L1-negative tumors occasionally benefit from anti-PD-1/PD-L1 therapy [19,24,35]. In the phase III OAK trial, atezolizumab resulted in OS improvement versus docetaxel, irrespective of the PD-L1 expression status in pretreated NSCLC patients [24]. These inconsistent predictive roles of PD-L1 expression may be due to the heterogeneous intratumoral distribution of PD-L1, dynamic change in PD-L1 expression, differential methods of PD-L1 detection, and cut-off values of PD-L1-positive tumor cells [36].

### 2.2. Tumor-Infiltrating Lymphocytes (TILs)

The central mechanism of anti-tumor immune responses is that activated cytotoxic T lymphocytes recognize tumor antigens specifically and eliminate cancer cells; therefore, TILs are a reasonable candidate to predict the efficacy of ICIs. Accumulating evidence has confirmed the prognostic impact of TILs in NSCLC patients, clarifying that increased levels of CD3 and CD8+ TILs are associated with better outcomes in NSCLC [37,38,39]. Regarding the response to ICIs and TILs, the CD8+ T-cell density at the invasive tumor edge was correlated with the response to anti-PD-1 treatment in melanoma patients [40,41]. Thus, not only PD-L1 expression but also TILs correlate with the efficacy of ICIs. Tumors are categorized into four subtypes according to the status of PD-L1 and TILs: type I (PD-L1+, TIL+), type II (PD-L1−, TIL−), type III (PD-L1+, TIL−) and type IV (PD-L1−, TIL+) [42]. Theoretically, type I tumors are most likely to benefit from anti-PD-1/PD-L1 blockade. Meanwhile, NSCLC patients occasionally display a type III tumor environment when PD-L1 is expressed constitutively on cancer cells through oncogenic signaling; they are unlikely to respond to single-agent anti-PD-1/PD-L1 treatment [42].

Other factors along with TILs may be important. Kinoshita et al. focused on the relationship between TILs, histological type, and smoking habit, showing that a low number of CD8+ T cells in non-adenocarcinoma, a high FOXP3/CD4 ratio in smokers with adenocarcinoma, and a low number of CD20+ B cells in non-smokers with adenocarcinoma were identified as independent prognostic factors in resected NSCLC [43]. It was also reported that the loss of HLA class I expression on tumor cells was linked to a reduced number of TILs [44]. Intriguingly, a low PD-1 incidence among CD8-positive cells was correlated with a prolonged survival in patients treated with nivolumab [45].

### 2.3. Tumor Mutational Burden (TMB) and Neoantigen Load

Mutations, genetic rearrangements, insertions, and deletions can generate neoantigens that are specific to the tumor relative to normal somatic cells. Tumors with a greater mutational burden could possess more neoantigens; thus, the patient may possess a larger repertoire of extant tumor-specific T cells [41]. Recently, the significance of TMB and neoantigen load in anti-tumor immunogenicity has gained increasing attention. The first reports on this issue have addressed the correlation between the mutational burden and the clinical benefit from anti-CTLA4 blockade in melanoma patients [46,47]. In NSCLC, Rizvi et al. first demonstrated that high nonsynonymous TMB (at a cut-off value of ≥178 mutations) was closely associated with favorable PFS in patients treated with anti-PD-1 therapy [48]. Additionally, they explored potential predictive biomarkers for the response to anti-PD-1/PD-L1 agents using targeted next-generation sequencing (NGS) in patients with NSCLC. Their analysis revealed that high TMB was correlated with a higher response rate and a durable clinical benefit [49]. While nivolumab did not improve PFS compared with platinum-based chemotherapy in untreated patients with advanced NSCLC with ≥5% PD-L1 expression [50], an additional analysis of this study showed that a PFS advantage of nivolumab over chemotherapy was observed in patients with high TMB [51]. Furthermore, a phase III study demonstrated that nivolumab plus ipilimumab significantly prolonged PFS compared with platinum-based chemotherapy in untreated NSCLC patients with high TMB [27]. These findings suggest that TMB could be a biomarker for ICIs.

Some unsolved issues persist concerning the clinical use of TMB as a biomarker for ICIs. First, the ranges of TMB were highly overlapped in responders and non-responders and the numerical cut-off of TMB that discriminates responders from non-responders has not been determined [47,48]. Second, the intratumoral heterogeneity of TMB may affect the prediction of the therapeutic response as the heterogeneity of the mutational load has been demonstrated in multi-regional samples within a lesion [36,52]. The clonal neoantigen load was correlated with OS in lung adenocarcinoma (LUAD), and the sensitivity to PD-1 and CTLA-4 blockade in patients with NSCLC and melanoma was enhanced in tumors enriched in clonal neoantigen [53]. As the predictive value of the mutation or neoantigen load has not been fully verified, further investigation of other aspects, such as self-antigen and insertion-and-deletion (INDEL)-derived tumor-specific neoantigen, is required. Studies have shown that the antitumor immune response conferred by T cells specific for both mutation-associated neoantigens and non-mutated tumor-associated self-antigen exists in melanoma patients [54]. The measurement of TMB is based on the amount of single-nucleotide variants (SNVs). In a study of renal cell carcinoma, which is generally responsive to ICIs but typically contains a modest number of mutations [55,56], INDEL mutations were a highly immunogenic mutational class that can trigger an increased abundance of neoantigens and greater mutant-binding specificity [57]. This study also showed that frameshift INDEL was significantly associated with the response to ICIs across three separate melanoma cohorts [57].

### 2.4. DNA Mismatch Repair (MMR) Deficiency

The loss of MMR genes is associated with a marked increase in TMB. The MMR-deficient genotype was identified through the detection of microsatellite instability (MSI). The relationship between this genetic subtype and the clinical activity of ICIs has been rigorously investigated mainly in colorectal cancer. The first hint of the predictive relevance of the MSI status arose from the finding that a durable response to anti-PD-1 treatment was observed in a patient with colorectal cancer, which is one of the cancers resistant to ICIs; this patient exhibited the MSI-high phenotype [58]. Consequently, a phase II trial examined the efficacy of pembrolizumab according to the MMR profile in patients with colorectal cancer, demonstrating that the ORR and the immune-related PFS rate at 20 weeks in MMR-deficient patients and MMR-proficient patients were 40% vs. 0% and 78% vs. 11%, respectively [59]. This evaluation was further expanded in MMR-deficient patients across 12 different tumor types, showing the marked findings that the ORR was 53%, including a complete response in 21%, and the estimated PFS and OS at 2 years were 53% and 64%, respectively [60]. In another phase II study of nivolumab, the ORR, disease control rate (DCR) and 1-year PFS rate of heavily treated patients with MMR-deficient or MSI-high colorectal cancer were 31.1%, 69% and 50%, respectively, and the median PFS was 14.3 months [61]. Furthermore, treatment with nivolumab plus ipilimumab yielded a more durable benefit for MMR-deficient patients; the CheckMate-142 trial showed that the investigator-assessed ORR, 1-year PFS rate and 1-year OS rate were 55%, 71% and 85%, respectively [62]. As for NSCLC, the role of the MMR or MSI status remains unclear due to the very low incidence of this genotype because MSI-high was detected in 0.3–0.8% of NSCLC patients [63,64].

## 3. The Efficacy of ICIs in Oncogenic-Driven NSCLC

Randomized phase III trials have indicated that patients carrying *EGFR* mutations exhibit less efficacy of anti-PD-1/PD-L1 treatment than those with wild-type *EGFR*, whereas *KRAS* mutations are likely to be predictors of favorable outcomes. Few studies have addressed the relationship between *ALK* rearrangement and clinical benefit from ICIs due to the small number of recruited patients with *ALK* rearrangement. The efficacy of ICIs in oncogenic-driven NSCLC is summarized in Table 1.

### 3.1. EGFR Mutations and ALK Rearrangements

Although several clinical trials have demonstrated the survival benefit of anti-PD-1/PD-L1 inhibitors over docetaxel in pretreated NSCLC patients, this has not been proven in *EGFR*-mutant populations [18,21,24]. Furthermore, clinical activity of atezolizumab in *EGFR*-mutated patients tended to be inferior compared to that in *EGFR*-wild type patients [65]. Consistently, in patients with stage III NSCLC receiving chemoradiation therapy, durvalumab did not improved PFS among *EGFR*-mutant patients [25]. A retrospective analysis showed that *EGFR* T790M-positivity was associated with unfavorable clinical outcome in patients treated with nivolumab following EGFR-TKI treatment [66]. Another retrospective analysis showed that anti-PD-1 monotherapy was ineffective in *EGFR*-mutant patients who had progressive disease with EGFR-TKI treatment, even in patients with ≥50% PD-L1 expression [67]. In addition, meta-analyses demonstrated that ICIs are less beneficial in *EGFR*-mutant NSCLC compared to chemotherapy [68] or EGFR-TKI therapy [69]. These findings indicate that ICI monotherapy has no therapeutic role for *EGFR*-mutant NSCLC. Meanwhile, the ATLANTIC trial reported contradictory findings that durvalumab exhibited clinical activity regardless of the status of *EGFR*/*ALK* alterations in heavily treated NSCLC with higher expression of PD-L1 [70]. Further investigation is warranted to evaluate the effect of ICIs for *EGFR/ALK*-positive NSCLC in larger-scale cohorts. In this respect, it is noteworthy that a randomized phase II trial (WJOG8515L) is currently ongoing to compare nivolumab with carboplatin plus pemetrexed chemotherapy in *EGFR*-mutant NSCLC patients who have developed resistance to EGFR-TKI due to mechanisms other than T790M [71].

Recently, the IMpower150 trial demonstrated that the addition of atezolizumab to bevacizumab plus carboplatin plus paclitaxel (ABCP) significantly improved PFS and OS compared to bevacizumab plus carboplatin plus paclitaxel (BCP) in advanced non-Sq NSCLC patients including those who had received TKIs, irrespective of *EGFR* or *ALK* genetic alteration status [72]. Of note, ABCP significantly improved PFS (HR, 0.41; 95% CI: 0.22–0.78) for the patients with *EGFR* exon19 deletion or L858R mutation compared to BCP in the subgroup analysis [73], showing that the ABCP therapy may be beneficial for *EGFR*-mutated NSCLC patients. Further studies of large-scale population will help to elucidate the therapeutic role of ICIs combined with bevacizumab plus chemotherapy in oncogenic-driven NSCLC after acquired resistance to TKIs.

### 3.2. KRAS Mutations

Among NSCLC patients with *KRAS* mutations, OS in the nivolumab group was significantly longer than that in the docetaxel group [18], whereas atezolizumab did not prolong OS compared with docetaxel [24]. A recent meta-analysis demonstrated that ICIs improved OS compared with docetaxel in pretreated NSCLC patients with *KRAS* mutations but not in those with *KRAS* wild-type [74]. Collectively, although *KRAS* mutations may predict the response or outcomes in NSCLC patients treated with anti-PD-1/PD-L1 blockade, its clinical relevance has not been fully proven due to the retrospective nature and insufficient examination of the genetic status.

## 4. PD-L1 Expression and Immunologic Features in Oncogene-Driven NSCLC

An association between PD-L1 expression and oncogenic drivers has been extensively explored in prospective studies and clinical practice. Recent experimental studies have elucidated the regulatory mechanisms of PD-L1 expression. Briefly, tumor cells can activate PD-L1 expression via the following mechanisms: (1) alterations of genes, including *EGFR*, *ALK* fusions, *KRAS*, *MYC*, *PTEN*, and p53; (2) exogenous inflammatory cytokines, such as interferon-γ; (3) *PD-L1* amplification; and (4) disruption of the 3′-untranslated region of the *PD-L1* gene [75]. Previous reports of PD-L1 expression according to status of oncogenic alterations are summarized in Table 2.

### 4.1. EGFR Mutations

Several preclinical studies have suggested that oncogenic EGFR signaling pathways upregulate PD-L1 expression. This issue was first addressed by Akbay et al., who demonstrated that forced expression of mutant EGFR in bronchial epithelial cells induced PD-L1 and that PD-L1 expression was reduced by EGFR inhibitors in *EGFR*-mutant NSCLC cell lines [76]. Other studies indicate that ERK/c-Jun [77] or NF-κB [78] is involved in mutant EGFR-mediated PD-L1 upregulation.

The positive correlation between *EGFR* mutations and PD-L1 overexpression has also been observed in NSCLC tumors. Azuma et al. showed that the presence of *EGFR* mutations was associated with high expression of PD-L1, independent of other characteristics [79]. D’Incecco et al. showed the consistent finding that the presence of *EGFR* mutations was linked to a PD-L1-positive status [80]. In contrast, several studies indicate no significant relationship existed between *EGFR* mutation status and PD-L1 expression [81,82,83]. PD-L1 positivity was significantly lower in *EGFR*-mutant LUADs than in those with wild-type *EGFR* [84], and PD-L1-positive LUADs carrying *EGFR* mutations were observed in only 9% of all *EGFR*-mutant LUADs [85]. Concordantly, recent meta-analyses have revealed that *EGFR* mutations were associated with decreased expression of PD-L1 [86,87,88]. Thus, from the perspective of a negative correlation between PD-L1 positivity and *EGFR* mutation status, it is likely that anti-PD-1/PD-L1 therapy has limited activity for *EGFR*-mutant NSCLCs.

Recent studies have provided some implications for immunological features other than PD-L1 expression that could confer the limited efficacy of ICIs for *EGFR*-mutant NSCLCs. NSCLC patients with *EGFR* mutations showed a shrinking proportion of PD-L1+/CD8+ TILs and decreased TMB [86]. Additionally, a low incidence of concurrent PD-L1 expression and CD8+ TILs in *EGFR*-mutant NSCLC tumors has been observed [85,89], possibly explaining the lower immune response in NSCLC patients with *EGFR* mutations.

### 4.2. ALK Rearrangement

The oncogenic capacity of *ALK* rearrangement to upregulate PD-L1 expression was first documented in *ALK*-translocated T-cell lymphoma [90]. In NSCLC, Ota et al. reported that forced expression of echinoderm microtubule-associated protein-4 (EML4)-ALK oncoprotein in Ba/F3 cells increased PD-L1 expression, whereas endogenous PD-L1 expression in *EML4-ALK*-positive NSCLC cells was attenuated by the ALK inhibitor alectinib and by siRNA-mediated ALK knockdown [91]. Hong et al. also observed these results and further demonstrated that ALK fusion-mediated upregulation of PD-L1 induced the apoptosis of T cells in tumors and a dendritic cell-CIK cell co-culture system [92]. Furthermore, it has shown that the downstream activation of MEK-ERK and PI3K-AKT pathways, STAT3, and HIF-1α could mediate *EML4-ALK*-induced upregulation of PD-L1 [91,92,93].

Clinicopathological studies have indicated a relationship between *ALK* rearrangement and PD-L1 overexpression. A previous study investigated the PD-L1 expression by IHC in resected NSCLC tumors, revealing that the *ALK*-rearranged tumors exhibited higher PD-L1 expression than those with wild-type *ALK* [91]. Another study showed that *ALK* translocation was correlated with PD-L1 and PD-L2 expression in the resected LUADs [83]. Furthermore, Roussel et al. described a higher frequency of tumors combining positive PD-L1 expression and infiltration by intratumoral CD8+ T cells or PD-1+CD8+ T cells in *ALK*-rearranged LUADs compared to those with *EGFR* mutation or those with wild-type *EGFR*/*KRAS* and non-*ALK* rearrangement [94]. By contrast, meta-analyses have not shown significant correlations between *ALK* rearrangement and PD-L1 expression [87,88]. 

### 4.3. KRAS Mutations

Previous studies have shown that *KRAS* mutations induce PD-L1 overexpression through activation of its downstream pathways in NSCLC [95,96,97]. Sumimoto et al. demonstrated that siRNAs targeting *KRAS* or *ERK2* and the MEK inhibitor decreased PD-L1 expression in *KRAS*-mutant NSCLC with PD-L1 overexpression [95]. Consistent with their findings, Chen reported that forced expression of mutant KRAS in human immortalized bronchial cells increased PD-L1 expression, which was attenuated by *KRAS* siRNAs and the ERK inhibitor [96]. We also reported that siRNA-mediated mutant KRAS knockdown and pharmacological inhibitors of MEK and ERK reduced PD-L1 expression in NSCLC cells with *KRAS* mutations and PD-L1 overexpression [97]. In our study, the mRNA expression levels of *CD274* encoding PD-L1 varied greatly among *KRAS*-mutated NSCLC cell lines, implying that other mechanisms modulate PD-L1 expression in different *KRAS*-mutant NSCLCs.

Clinicopathological studies have indicated the positive correlation between *KRAS* mutations and PD-L1 expression in NSCLC [80,85,87,88,96,98,99,100]. Scheel et al. reported that *KRAS*-mutated NSCLC tumors exhibited a higher positivity of PD-L1 expression [98]. Several meta-analyses have revealed that *KRAS*-mutated tumors were significantly associated with higher levels of PD-L1 expression [87,88,99]. In addition, the smoking status was correlated with PD-L1 expression in *KRAS*-mutant NSCLC. Calles et al. suggested that smoking is likely to be associated with increased PD-L1 expression; PD-L1 expression is detected in 44% of current smokers, 20% of former smokers, and 13% of never smokers, and a higher intensity of PD-L1 expression was frequently observed in smokers with more pack-years [101].

Several experimental and translational studies have addressed distinct immunological features in *KRAS*-mutant NSCLC. Co-occurring genetic alterations in *STK11/LKB1* (KL subgroup) and *TP53* (KP subgroup), as well as *CDKN2A/B* inactivation coupled with low expression of TTF1 transcription factor (KC subgroup), define three major subgroups of *KRAS*-mutant LUAD with distinct biology and therapeutic vulnerabilities [102]. In this study, the KC subtype exhibited mucinous histology and suppressed mTORC1 signaling; the KL subtype expressed lower levels of immune markers, including PD-L1, and showed increased vulnerability to HSP90-inhibitor therapy; and the KP subtype showed higher levels of somatic mutations, inflammatory markers and immune checkpoint effector molecules and improved relapse-free survival. Previous studies evaluated the significance of *TP53* and *KRAS* mutations in LUAD and provide some notable findings: (1) co-occurring mutation in *TP53* and *KRAS* showed the highest PD-L1 expression, the highest proportion of concurrent PD-L1 expression and CD8+ T-cell infiltration, decreased expression of some other non-PD-L1 immune inhibitory checkpoints, such as lymphocyte activating 3 (LAG-3), and higher mutational load; (2) *KRAS* mutation manifested as various defects in DNA repair, including MMR-related genes; (3) *TP53* mutations increased the mutation frequencies of *POLE*, which is associated with the disruption of exonuclease activity required for DNA proofreading and leads to high TMB; and (4) the patients with *KRAS* or *TP53* mutations, especially those with co-occurring *TP53/KRAS* mutations, showed a marked clinical benefit to PD-1 inhibitors [103,104,105]. Zdanov et al. suggested that mutant KRAS induced the secretion of IL-10 and transforming growth factor-β1 (TGF-β1) and contributed to the induction of regulatory T cells [106]. The most recent clinicopathological study demonstrated that *KRAS*-mutant LUAD specimens exhibited higher expression of PD-L1 in malignant cells and of B7-H3, T-cell immunoglobulin mucin family member 3 (TIM3), and indoleamine 2, 3-dioxygenase-1 (IDO-1) in stromal tumor-associated inflammatory cells than wild-type [100].

### 4.4. BRAF Mutations

The proto-oncogene *BRAF* is mutated in 2% to 4% of LUAD [107], and approximately half of *BRAF*-mutated NSCLCs have non-V600E mutations [108,109]. Given that the therapeutics recently approved for *BRAF*-mutant NSCLC only target the V600E mutation, treatment strategies for non-V600E *BRAF*-mutated NSCLC should be urgently developed, and immunotherapy may contribute to establishing the strategies.

Significant associations between PD-L1 positivity and *BRAF* mutation have been observed in colon cancer [110,111,112] and melanoma [113]. Owing to the rarity of *BRAF*-mutant NSCLC, little is known about the immune response and immunological characteristics in such populations. A retrospective analysis in LUAD patients showed that *BRAF* mutation was significantly associated with a low density of intratumoral CD8+ T cells and high neutrophils density [114]. Recently, Dudnik et al. retrospectively analyzed the immunological features in NSCLCs with *BRAF* V600E mutation (group A) and those with *BRAF* non-V600E mutation (group B) [115]. This study suggests that *BRAF* mutation is associated with a high level of PD-L1 expression, low/intermediate TMB, and a microsatellite-stable status. This study also described that the ORRs with ICIs were 25% and 33% in groups A and B, respectively, and the median PFS rates with ICIs were 3.7 and 4.1 months in groups A and B, respectively, indicating that ICIs may have favorable anti-tumor activity for both *BRAF* V600E and *BRAF* non-V600E mutant NSCLC.

## 5. Conclusions and Future Directions

Although molecular targeted therapies have been established in some subsets of patients with oncogene-driven NSCLC, the role of immunotherapy remains unclear. Previous studies have suggested that the status of driver oncogenes in NSCLC tumors seems predictive for the efficacy of immunotherapy, but the predictive roles have not been determined. Given that targeting NSCLCs harboring *KRAS* mutations or *BRAF* non-V600E mutations remains challenging, immunotherapy may play a pivotal role in patients carrying these genotypes. Thus, further investigation into the relationship between oncogenic drivers and immunological backgrounds should be required. Accumulating evidence has speculated that a single biomarker may not fully predict the clinical activity of immunotherapy. Thus, exploring the combination of biomarkers for patient selection to maximize the therapeutic benefits or minimize medical and economic loss would be a cornerstone of the development of cancer immunotherapy in the future.

## Figures and Tables

**Table 1:** The efficacy of immune checkpoint inhibitors (ICIs) in oncogenic-driven non-small cell lung cancer (NSCLC).

Reference	Agents	*EGFR*-Mutant			*ALK*-Rearranged			*KRAS*-mutant		
		Patients Included, *n*	PFS HR [95% CI]	OS HR [95% CI]	Patients Included, *n*	PFS HR [95% CI]	OS HR [95% CI]	Patients Included, *n*	PFS HR [95% CI]	OS HR [95% CI]
CheckMate 057 [18]	Nivolumab vs. Docetaxel	82 (14%)	1.46 [0.90–2.37]	1.18 [0.69–2.00]	21 (4%)	NA	NA	62 (11%)	0.82 [0.47–1.43]	0.52 [0.29–0.95]
KEYNOTE 010 [21]	Pembrolizumab vs. Docetaxel	86 (8%)	1.79 [0.94–3.42]	0.88 [0.45–1.70]	8 (0.7%)	NA	NA	NA	NA	NA
OAK [24]	Atezolizumab vs. Docetaxel	85 (10%)	NA	1.24 [0.71–2.18]	2 (<1%)	NA	NA	59 (7%)	NA	0.71 [0.38–1.35]
PACIFIC [25]	Durvalumab vs. Placebo	43 (6%)	0.76 [0.35–1.64]	NA	NA	NA	NA	NA	NA	NA
Meta-analysis by Lee, et al. [68]	Nivolumab or Pembrolizumab or Atezolizumab vs. Docetaxel	186 (10%)	NA	1.05 [0.70–1.55]	NA	NA	NA	NA	NA	NA
Meta-analysis by Sheng, et al. [69]	Nivolumab or Pembrolizumab or Atezolizumab vs. Docetaxel	NA	1.57 [1.07–2.31]	1.05 [0.69–1.59]	NA	NA	NA	NA	NA	NA
Meta-analysis by Kim, et al. [74]	Nivolumab or Atezolizumab vs. Docetaxel	NA	NA	NA	NA	NA	NA	148 (29%)	NA	0.64 [0.43–0.96]

Abbreviation: PFS, progression-free survival; OS, overall survival; HR, hazard ratio; 95% CI, 95% confidential interval; NA, not available.

**Table 2:** Programmed death-ligand 1 (PD-L1) expression according to status of oncogenic alterations.

Reference	Methods	Antibody Company (Clone)	Cutoff	Driver Genes	Sample Size (Mut. vs. Wild)	PD-L1 Positivity (Mut. vs. Wild)	OR [95% CI] (Mut. vs. Wild)	*p* Value
Azuma, et al. [79]	IHC	Lifespan Biosciences	>Median value of H-score (30)	*EGFR*	57 vs. 107	NA	25.4 [2.9–47.9]	0.027
Takada, et al. [84]	IHC	Spring Bioscience (SP142)	>1% or >5% with positive cells	*EGFR*	112 vs. 123	18% vs. 36% (1% cutoff) 7% vs. 26% (5% cutoff)	NA	0.0021 (1% cutoff) <0.0001 (5% cutoff)
Dong, et al. [86] (pooled analysis)	IHC	Various	Various	*EGFR*	1050 vs. 2233	NA	1.79 [1.10–2.93] (wild vs. mut.)	0.02
Chen, et al. [96]	IHC	Cell Signaling (E1L3N)	NA	*KRAS*	19 vs. 38	H-score (median) 60 vs. 30	NA	0.042
Scheel, et al. [98]	IHC	Generated by Dr. Lieping Chen (5H1)	>1% with positive cells	*KRAS*	55 vs. 68	42% vs. 22%	2.5 [1.2–5.6]	0.018
D’Incecco, et al. [80]	IHC	Abcam (ab58810)	>5% with at least moderate staining	*EGFR*	56 vs. 69	71% vs. 41%	NA	0.001
				*ALK*	10 vs. 115	60% vs. 54%	NA	NS
				*KRAS*	29 vs. 96	52% vs. 55%	NA	0.84
Yang, et al. [81]	IHC	Proteintech Group	≥5% with at least moderate staining	*EGFR*	97 vs. 66	44% vs. 33%	NA	NS
				*ALK*	3 vs. 160	67% vs. 39%	NA	NS
				*KRAS*	8 vs. 155	63% vs. 39%	NA	NS
				*BRAF*	7 vs. 156	57% vs. 39%	NA	NS
Koh, et al. [83]	IHC	Cell Signaling (E1L3N)	≥10% with at least moderate staining	*EGFR*	228 vs. 171	56% vs. 62%	NA	NS
				*ALK*	23 vs. 474	78% vs. 58%	NA	NS
				*KRAS*	25 vs. 174	64% vs. 56%	NA	NS
Huynh, et al. [85]	IHC	Cell Signaling (E1L3N)	≥5% with positive cells	*EGFR*	54 (mut.)	9% (mut.)	0.24 [0.05–1.06]	NS
				*ALK*	4 (mut.)	25% (mut.)	0.22 [0.00–14.77]	NS
				*KRAS*	108 (mut.)	46% (mut.)	1.67 [0.64–4.34]	NS
Zhang, et al. [88] (meta-analysis)	IHC	Various	Various	*EGFR*	1560 vs. 2787	30% vs. 34%	0.61 [0.42–0.90]	0.01
				*ALK*	69 vs. 1967	42% vs. 35%	1.02 [0.61–1.71]	NS
				*KRAS*	341 vs. 1887	29% vs. 35%	1.34 [1.00–1.79]	NS
Lan, et al. [87] (meta-analysis)	IHC	Various	Various	*EGFR*	4891 (total)	NA	0.64 [0.45–0.91]	0.014
				*ALK*	3050 (total)	NA	1.40 [0.91–2.15]	NS
				*KRAS*	3167 (total)	NA	1.45 [1.18–1.80]	0.001
Yang, et al. [99]	IHC	Various	Various	*EGFR*	908 vs. 1552	37% vs. 31%	0.74 [0.52–1.06]	NS
				*ALK*	57 vs. 1556	40% vs. 33%	1.02 [0.75–1.38]	NS
				*KRAS*	365 vs. 1689	32% vs. 32%	1.26 [1.06–1.50]	0.010

Abbreviation: mut., mutant; OR, odds ratio; 95% CI, 95% confidential interval; IHC, immunohistochemistry; NA, not available; NS, not significant (*p* < 0.05).
